# A Critical Perspective on NeuroRights: Comments Regarding Ethics and Law

**DOI:** 10.3389/fnhum.2021.703121

**Published:** 2021-10-25

**Authors:** Diego Borbón, Luisa Borbón

**Affiliations:** ^1^The Latin American Observatory of Human Rights and Enterprises, NeuroRights Research Line, Universidad Externado de Colombia, Bogotá, Colombia; ^2^Faculty of Engineering, University of Los Andes, Bogotá, Colombia

**Keywords:** NeuroRights, neuroethics, enhancement, free will, mental privacy, bias, personal identity, neurolaw

In this article, we present comments on the five NeuroRights proposed by the NeuroRights Initiative. This analysis seeks to offer some critical views regarding challenges in free will, enhancement, identity, algorithmic bias, and privacy. Our paper focuses on some conceptual, practical, ethical and logical problems that need to be consider in order to determine whether those NeuroRights should become a global policy. We believe that, although they constitute an innovative proposal from neuroethics and neurolaw, it is possible to glimpse some concerns that cast doubt on the convenience of incorporating them.

## Neuroright to Free Will

The NeuroRights Initiative defines it as: “individuals should have ultimate control over their own decision making, without unknown manipulation from external neurotechnologies” (NeuroRights Initiative, 2021). Nevertheless, it seems conceptually problematic to propose a NeuroRight under this label. Free will is a fundamental problem that has been haunting philosophers for more than two millennia (Harris, 2012; Kane, 2012; O'Connor and Franklin, 2021). The debate concerning free will is far from being a peaceful matter. At least for now, there are two main positions: compatibilism and incompatibilism (Muñoz, 2012). On one side, compatibilism is the thesis that it is metaphysically possible that determinism is true, and people have free will (McKenna and Pereboom, 2016; van Inwagen, 2017). On the other side, hard incompatibilism is the thesis that our actions are either deterministic or truly random events and both possibilities exclude free will and moral responsibility (Pereboom, 2003).

Furthermore, if neuroscience has allegedly created a case against free will, since the proposed experiments (Libet et al., 1983; Haggard and Eimer, 1999; Soon et al., 2008, 2013; Fried et al., 2011), it seems contradictory to suggest the creation of a “Neuro” Right to free will. In this perspective, trying to elevate “Free will” as a category of human rights seems deeply complicated in conceptual terms (Muñoz, 2019; Borbón et al., 2020). In this sense, we envision that a right, under the philosophical baggage of free will, should not be incorporated. If we aim to protect consent to the use of neurotechnologies, this protection should be included within the current right to informed consent.

## Neuroright to Equal Access to Mental Augmentation

Another concern that should be addressed is the ethical and practical repercussions of promoting access to enhancement. The NeuroRights Initiative proposes to establish guidelines regulating the development and applications of enhancement neurotechnologies based on the principle of justice and guarantee equality of access to all citizens (Yuste et al., 2017; NeuroRights Initiative, 2021).

Right now, neurotechnological research is being conducted in this area toward enhancing the user's cognitive capacities in a variety of tasks (Valeriani et al., 2017; Cinel et al., 2019; Belkacem et al., 2020; Kaimara et al., 2020). However, we find it problematic to create a new right that promotes access to enhancement technologies, as this could lead to possible transhumanist applications that should be treated with caution.

In that direction, as the alteration of the human nature^1^ becomes a social fact, the freedom of those who do not wish to improve could be significantly affected. This would create new social, labor and academic standards that would forge pressure on people who could not bear to be treated as inferior in these fields compared to their enhanced peers. The foregoing enters in contradiction with the proposed neuro-right to free will in the sense that people would not be giving consent free of vices but falling in front of the new social norms created with this new right (Borbón et al., 2021).

Furthermore, a NeuroRight to enhancement, if not adequately limited, may imply that the State should assume a new financial burden to provide and subsidize these types of technologies to vulnerable groups of citizens. Considering that the majority of public health systems exclusively finance therapeutic interventions, the State should not be assuming this new burden to guarantee enhancement with public resources. This is what seems to be happening in Chile, since the “Neuroprotection Bill,” promoted by the NeuroRights Initiative, provides textually in article 10 that: “The State will guarantee the promotion and equitable access to advances in neurotechnology and neuroscience” (Senado de Chile, 2020). Unfortunately, the way the text was drafted does not provide any clarity on the scope, limits and obligations of the proposal, raising more questions than answers.

In addition, this right could be considered obsolete in developing countries, such as Latin American ones, as some of them can-not even provide access to the most basic needs, such as nutrition or health care, and the guarantee of human rights (Cheru, 2016; Ezzati et al., 2018; Macarayan et al., 2018). Consequently, the gaps between developed and developing countries will widen, increasing power asymmetries. In that sense, an ethical proposal should be guided toward the extensive regulation of enhancement applications, with new laws and international treaties. Failure to do so would possibly involve leaving the door open to unlimited corporate interests for those companies that develop neurotechnologies, since it would be financing, with public funds, the numerous acquisitions of technologies whose purposes are not therapeutic, nor for public health, in the name of a new ambiguous human right (Borbón et al., 2021).

On the other hand, it is relevant to adapt this proposal to the various cultural and social contexts, especially those of Latin American countries. The foregoing in the sense that normalizing the possibility of mental augmentation, and even making it a human right, may go against religious precepts and cosmologies of indigenous groups, where the modification of human nature and the intimate interaction with neurotechnologies would not necessarily be viewed favorably (Borbón et al., 2021). In this sense, we propose not to incorporate this new human right as a subjective faculty to claim mental augmentation.

## Neuroright to Personal Identity

The NeuroRights Initiative defines personal identity as “Boundaries must be developed to prohibit technology from disrupting the sense of self” (NeuroRights Initiative, 2021). Nonetheless, as it is assumed that the use of neurotechnology with enhancement purposes is inevitable and that equitable access should be promoted, it is necessary to assess that any intervention in the brain might cause some alteration in the mind and potentially threatens personal identity (Kraemer, 2013; Klein et al., 2015; Mackenzie and Walker, 2015; Iwry et al., 2017; Gilbert et al., 2019).

In that sense, a NeuroRight to cognitive enhancement enters in a problematic antinomy with the right to personal identity. Depending on the definition of self, identity and authenticity, prohibiting technology from altering these personal traits may imply prohibiting neurotechnologies in general. Precisely, one of the great challenges is drawing the limits in the definition of personal identity and its disruption. Furthermore, it is not simple to state a priori in which way neurotechnologies can threaten the self in order to regulate them. In general, we should certainly strive toward establishing some kind of precautionary principle when considering neurorights (Inglese and Lavazza, 2021). In that sense, a broader discussion needs to take place before establishing this right.

## Neuroright to Protection From Algorithmic Bias

As technology advances and artificial intelligence algorithms become more intertwined with our daily lives and our mental data, the attention to the potential harm of algorithmic biases has dramatically increased. In this scenario, the NeuroRights Initiative (2021) proposes to establish explicit countermeasures against bias, like employing input from relevant user groups into the training datasets. Although we believe the intention behind the proposal is positive, some aspects need to be taken into consideration.

First, the race against bias should go beyond treating all algorithmic biases as something we shall aspire to eliminate. As mentioned by Danks and London (2017) many of these biases are neutral or can even be beneficial in our efforts to achieve our diverse goals. These authors expose different types of algorithmic biases and highlight how in some cases, an algorithmic bias can be used to mitigate the effect of another and contribute to the system performing according to the relevant ethical and legal standards. In this sense, saying that we must eliminate all biases is an oversimplification of the complex problem they impose.

Moreover, this right mentions the need to include input from user groups to foundationally address bias. In the technological sector, sharing the data used to train intelligent systems should be a baseline definition of transparency, from which others can actively work toward improving the accountability of the algorithms (Buolamwini and Gebru, 2018). However, assembling a nonbiased dataset is not always possible or sustainable. Usually, the data and algorithms deployed commercially are protected by copyright and patents. This turns the process of collecting representative datasets into an expensive and demanding task, among many things, because the sources of information are limited due to privacy issues. In this case, we are not necessarily against establishing this right, but the considerations mentioned need to be considered as well as further study on the matter.

## Neuroright to Mental Privacy

As the gap between our mental information and technology narrows, data privacy issues are gaining higher relevance for neurotechnologies. The fact that this sensitive data unveils the intentions and internal states of its users, demands the need for protection by raising awareness and using advanced security techniques (Yuste et al., 2017). In this sense, the NeuroRights Initiative (2021) defines that according to mental privacy: “any data obtained from measuring neural activity should be kept private.”

In this regard, techniques such as federated learning are being developed to protect and secure valuable information. These strategies aim to provide local data processing so that this information can be used by AI algorithms while maintaining the integrity and privacy of the users. Nowadays, reliable federated learning systems are being deployed on mobile networks (Kang et al., 2020), but we are still far from being able to use them in a practical matter with our neural data. Ideally, these promising techniques will work using brain data in the near future, but in the meantime, we can't just stand with folded hands and keep our mental data a secret.

Furthermore, this right can adversely affect the need to protect the users from algorithmic biases. While we strive to secure our individual data, it will be increasingly arduous to obtain databases that are representative enough of the collectives; therefore, making it difficult to develop fairer algorithms without potentially harmful biases. Moreover, this restriction would greatly limit the innovation and development of neurotechnologies as the value of these devices comes from being able to make robust models by comparing data of numerous individuals.

## Are Neuro-Rights Necessary?

Here we have shown the conceptual inconvenience of free will, and the practical and ethical issues involving enhancement, privacy, identity and bias. In addition to the antinomies that may arise, it is relevant to question the need to create a new category of human rights. Most national and international legal systems already protect freedom, consent, equality, integrity, privacy, and information. We view with skeptical eyes the advisability of creating a new category of rights. Moreover, considering that the creation of new rights implies a general and not very exhaustive description, we do not see that they can effectively regulate the neurotechnological advance. We propose that it is necessary to prepare justice operators to adequately interpret constitutional rights considering the challenges presented by neurotechnologies. In the same way, clear, extensive, and sufficient legal and international regulations must be established to satisfactorily address the limits to neurotechnologies.

All things considered; we suggest that the proposal of the neuro-rights should be extensively reviewed. Also, the scope and limits of each right should be adequately analyzed before attempting to incorporate them. For this, we propose that the academic and political discussion scenarios be expanded, especially by integrating the views of more Latin American countries, in addition to Chile. Our comments, of course, do not claim to be absolute truth, nor can we answer with certainty whether NeuroRights should become global politics and how, but we hope that these brief considerations will serve to enrich the discussion.

## Author Contributions

All authors listed have made a substantial, direct and intellectual contribution to the work, and approved it for publication.

## Conflict of Interest

The authors declare that the research was conducted in the absence of any commercial or financial relationships that could be construed as a potential conflict of interest.

## Publisher's Note

All claims expressed in this article are solely those of the authors and do not necessarily represent those of their affiliated organizations, or those of the publisher, the editors and the reviewers. Any product that may be evaluated in this article, or claim that may be made by its manufacturer, is not guaranteed or endorsed by the publisher.

## Footnotes

^1^The concept of human nature is, without a doubt, a difficult term to define or to provoke consensus among scholars. In that sense, we want to guide the conversation above all to the discussions around posthumanism. Fukuyama (2002) is a defender of concepts such as human nature that he defines as: “human nature is the sum of the behavior and characteristics that are typical of the human species, arising from genetic rather than environmental factors” (p.130). Pepperell (2003), defender of posthumanism, maintains that: “the posthuman era begins when we no longer find it necessary, or possible, to distinguish between humans and nature; a time when we truly move from the human to the posthuman condition of existence” (p. 161).
